# Stress coping strategies in parents of children with central coordination disorders undergoing Vojta therapy: a two-month prospective observational study

**DOI:** 10.3389/fpsyg.2026.1875366

**Published:** 2026-07-01

**Authors:** Kinga Strojek, Dorota Wójtowicz, Małgorzata Stefańska, Joanna Rymaszewska, Joanna Kowalska

**Affiliations:** 1 Faculty of Physiotherapy, Wroclaw University of Health and Sport Sciences, Wroclaw, Poland; 2 Department of Clinical Neurosciences, Faculty of Medicine, Wroclaw University of Science and Technology, Wroclaw, Poland

**Keywords:** central coordination disorders, emotional state, physical activity, stress coping strategies, Vojta therapy

## Abstract

**Background:**

Parents of children with illnesses employ a variety of coping strategies. These strategies can play a crucial role in modulating the level of parental stress and mood. The study aimed to analyse stress-coping strategies used by parents of children with central coordination disorders (CCD) undergoing Vojta therapy over a period of 2 months.

**Methods:**

A total of 57 participants who completed questionnaires before starting their child’s therapy (T1) and after 2 months (T2), with a mean age of 32.8 (±4.6) were assessed. The Perceived Stress Scale (PSS-10), the Patient Health Questionnaire (PHQ-9), the Brief COPE Inventory (Mini-COPE) and a self-designed questionnaire were used.

**Results:**

At T1 and T2, the study group of parents most frequently used strategies such as Active Coping and Seeking Emotional Support. Female participants more often than males chose emotional and religious strategies. At T1 and T2, physically active parents significantly more often used the strategy Active Coping and Acceptance (respectively) and had significantly lower stress levels and better mood, compared with inactive parents.

**Conclusion:**

Planned interventions for parents of children with CCD should focus on encouraging them to engage in physical activity and strengthening personal resources by fostering adaptive coping strategies, particularly problem-oriented coping strategies to improve their psychological well-being.

## 1 Introduction

The diagnosis of a developmental disorder (DD) in a child is associated with a significant psychological burden for parents (Einfeld and Emerson, 2008). Raising a child who requires special care constitutes a major challenge and involves the need for intensive support from caregivers in meeting the child’s basic physical, social, and emotional needs, as well as in striving to ensure a good quality of life (Petry and Maes, 2007). Consequently, this situation may lead to a substantial psychosocial burden for parents, manifested, among others, in depressive symptoms, marital conflicts, and deterioration of physical health (Samadi et al., 2020; Sakkalou et al., 2018; Jean et al., 2018; Theule et al., 2013).

Parents of children with DD function within a specific psychosocial context characterized by elevated emotional and organizational burdens. A child’s illness and/or developmental disorders represents an exceptionally stressful situation. Previous research has consistently shown that parents of children with DD experience significantly higher levels of parental stress compared with parents of typically developing (TD) children or those from other clinical groups (Theule et al., 2013; Allen et al., 2013; Barroso et al., 2018; Pastor-Cerezuela et al., 2016; Karaivazoglou et al., 2018; Hou et al., 2018; Huang et al., 2019; Takuri, 2017; Hayes and Watson, 2013). In particular, they face greater caregiving demands and report not only elevated stress but also heightened levels of general anxiety, anticipatory anxiety related to their child’s future, as well as intensified symptoms of depression (Cuzzocrea et al., 2016; Antonopoulou et al., 2020; Bujnowska et al., 2019; Scherer et al., 2019).

Folkman and Lazarus (Folkman and Lazarus, 1985) define stress as a dynamic interaction between the individual and the environment, in which the individual evaluates whether situational demands exceed their available resources. In this framework, coping is understood as the cognitive and behavioral efforts undertaken to manage, tolerate, or minimize internal or external demands appraised as taxing or exceeding the individual’s adaptive capacities. According to the classification proposed by Lazarus and Folkman (Folkman and Lazarus, 1985), then Endler and Parker (Endler and Parker, 1990), three primary types of coping strategies can be distinguished: problem-focused (task-oriented), emotion-focused strategies and avoidance-oriented strategies. The former are directed toward actively solving the problem or modifying the stressful situation. The second are aimed at emotional regulation and the reduction of psychological tension, whereas the third are less effective and can provide short-term relief. These processes are of particular importance in the context of parenting under conditions of high caregiving burden (Aratti and Zampini, 2024).

Parental stress has a significant impact on the functioning of both parents and children, being associated with increased emotional burden in caregivers, reduced quality of parenting attitudes, and the occurrence of behavioral difficulties and psychosocial developmental disturbances in children (Fang et al., 2022). This phenomenon is particularly evident among parents of children undergoing rehabilitation with the Vojta method, in whom high levels of stress and the presence of depressive symptoms have been confirmed in published pilot studies (Strojek et al., 2022).

The Vojta method is a neurophysiological approach used in the diagnosis and therapy of motor disorders in infants and children (Vojta, 1970). It is based on the concept of reflex locomotion, according to which specific movement patterns are innate within the central nervous system (CNS) and can be activated through targeted external stimuli (Banaszek, 2010). It is applied in many cases, including infants and children with DD, such as intellectual disability, autism, developmental delay, or genetic syndromes. It is also a method frequently used among children with central coordination disorders (CCD). The CCD is a diagnostic term commonly used in clinical practice in Central Europe to describe early motor coordination difficulties in infants and young children. Gross motor impairments, manifested primarily by abnormalities in muscle tone and the development of postural reactions, can be identified during the early stages of motor development. According to Vojta’s diagnostic approach, signs of CCD observed during the first months of life indicate abnormal development of postural reactions. The process of CCD detection is particularly important, as it allows for the evaluation of the dynamics and direction of developmental changes in children (Vojta, 1970).

Developmental Coordination Disorder (DCD) is one of the most common yet least studied neurodevelopmental disorders. DCD corresponds to the condition referred to as “specific developmental disorder of motor function”, defined as a delay in the development of motor coordination that cannot be explained by intellectual disability or another specific neurological condition (Blank et al., 2012). Children with CCD presented motor difficulties characteristic of Developmental Coordination Disorder (DCD) as described in DSM-5, which constituted the basis for the implementation of Vojta therapy.

Vojta therapy places high demands on parents, who act as active co-therapists and are responsible for its daily implementation in the home environment. For the therapy to be effective, it should be performed several times a day (most often 3–4 times), with each session typically lasting from 5 to 20 min. The effectiveness of the therapy depends not only on the underlying condition but also on the intensity, frequency, and accuracy of the exercises performed. The therapy has a combined character and includes both regular visits to a therapist (usually 1–2 times per week) and systematic work at home. The therapy program, its frequency, and any necessary breaks are continuously adjusted depending on the child’s progress, and the overall treatment process may last from several weeks to many months, and in some cases even years (Öhman et al., 2010; Castilla et al., 2023; Jung et al., 2017; Roșca et al., 2022).

Previous studies indicate high effectiveness of this method (Khavanskaya et al., 2020; Czajkowska and Kaptur, 2016); however, it continues to raise considerable controversy among both researchers and parents (Wójtowicz et al., 2007; Dytrych, 2008). The main source of this controversy is the fact that the therapy may evoke crying and visible discomfort in the child, which is sometimes interpreted as excessive intensity or even “brutality” of the method (Sadowska et al., 2001).

However, it should be emphasized that existing studies do not confirm a negative impact of Vojta therapy on children’s emotional development. Moreover, crying in infants represents a natural and non-specific form of communication, occurring in many everyday situations, and its presence during therapy does not necessarily indicate harmful effects- in a forced situation, the child defends itself against discomfort and exertion, and therefore may respond with loud crying (Frohlich, 1998) and the child’s crying during therapy has also been identified as the main barrier to its continuation (San-Martín-Gómez et al., 2026).

In particular, children with CCD often exhibit hypersensitivity to tactile stimuli and changes in body position, which may intensify emotional reactions regardless of the therapeutic method used (Dytrych, 2008; Sowa, 2004; Sadowska, 2001). Therefore, the controversy surrounding Vojta therapy appears to stem primarily from the interpretation of the child’s behavior during therapy rather than from clear evidence of its negative effects. This may constitute an additional source of stress for parents, who in Vojta therapy act as active co-therapists of their child. Stress, the child’s discomfort during therapy, emotional fluctuations, fatigue, and high self-imposed demands constitute some of the main challenges faced by parents implementing Vojta therapy in the home setting (García-Bravo and Martínez-Piédrola, 2023; Verhaegh and Nuijen, 2022; Medina-Mirapeix and Lillo-Navarro, 2017). In this context, exploring parental coping strategies appears essential for optimizing therapeutic outcomes and minimizing the psychosocial burden associated with long-term, intensive rehabilitation.

Parents of children with DD employ a variety of coping strategies to counteract the psychological burden associated with the need for long-term and intensive caregiving (Shilubane and Mazibuko, 2020; Wong et al., 2020). The effectiveness of these strategies plays a crucial role in modulating the level of parental stress and shaping family quality of life. Empirical studies indicate that coping strategies significantly influence the severity of mental health problems across different populations (Carlson and Miller, 2017). However, findings regarding parents of children with DD remain inconclusive. Some studies show that parents—particularly mothers—seek social support more frequently than parents of typically developing children (Craig et al., 2020), whereas other research has found no significant differences between these groups (Antonopoulou et al., 2020). It has also been demonstrated that the use of emotion-focused coping strategies in this population is associated with reduced psychological well-being and a higher perceived caregiving burden, while active avoidance contributes to increased stress and mental health symptoms (Medina-Mora et al., 2019; Shokoohi-Yekta et al., 2015). Moreover, the child’s challenging behaviors may further exacerbate parental fatigue and indirectly intensify the use of less effective strategies, leading to higher levels of stress and anxiety (Antonopoulou et al., 2020).

At the same time, studies indicate that parents of children with DDs are less likely to seek emotional support from others and less inclined to turn to others for sympathy or understanding compared with parents of TD children (Bawalsah, 2016; Halstead et al., 2018; Vernhet et al., 2019). As a result, the intensity of avoidance strategies is lower among them, since their primary priority remains caregiving. Research findings also demonstrate that parents of children with developmental disorders possess a personal resource in the form of constructive coping strategies—both in general situations and in those specifically related to the care and upbringing of a child with DD (Alós et al., 2022).

A two-month observation period reflects the early, intensive phase of therapy, during which parents adapt to the role of active co-therapists. In pediatric rehabilitation models, parents may act as primary implementers of interventions (An and Palisano, 2014), and therapeutic programs often require high intensity and many hours of practice (Gordon, 2011). This is associated with the need to reorganize professional and family life, as well as with a substantial time burden (McConnell et al., 2015). Moreover, adherence to therapeutic recommendations has been shown to contribute to increased parental stress as a secondary effect of therapy (Peplow and Carpenter, 2013). Therefore, this period may constitute a critical stage for the development and modification of coping strategies related to stress arising from therapeutic and caregiving demands.

Previous studies suggest that individual and psychosocial factors—such as sex and level of physical activity—may significantly influence both the perception of stress and the coping strategies employed (Davidson et al., 2024; van der Lubbe et al., 2025). At the same time, findings regarding differences in coping strategies themselves remain inconclusive, especially among parents of children with developmental disorders (Haripriya and Deepthi, 2023; Xu and Zhang, 2025). Inconsistencies in the existing findings justify the need for further analyses, including a stratified approach that may better capture variability in stress responses depending on key individual characteristics, especially given that available data on parents of children with CCD undergoing rehabilitation using the Vojta method—particularly regarding the coping strategies they employ—remain limited.

Therefore, the aim of the present study was to conduct a detailed analysis of stress-coping strategies used by parents of children with CCD undergoing Vojta therapy in the context of perceived stress levels and mood over a period of 2 months. The study focuses on parents as co-therapists, taking into account factors that may influence stress levels (including sex, physical activity and mood), which may be of particular importance for the effectiveness of therapy, as well as on coping mechanisms that may play a crucial role in the course and outcomes of treatment.

Based on the existing literature, it was hypothesized that: (1) parents of children with CCD undergoing Vojta therapy would predominantly use specific coping strategies, such as active coping and seeking support; (2) the use of some coping strategies can be characteristic in a group of parents with high levels of perceived stress and depressive symptoms; and (3) coping strategies and psychological outcomes would differ depending on individual factors such as sex and physical activity level.

## 2 Materials and methods

### 2.1 Study design

This prospective observational study was conducted in centers providing Vojta therapy for children, including the Diagnostic and Rehabilitation Center of the “Promyk Słońca” Foundation and the “Creator” Non-Public Health Care Institution in Wrocław, between November 2023 and April 2025.

The study was designed and implemented based on a previously published pilot study in the International Journal of Environmental Research and Public Health in 2022 (Strojek et al., 2022).

The study involved a two-month observation of parents of children with central coordination disorders (CCD), corresponding to the early, intensive phase of Vojta therapy. Parents, who consecutively reported to the above-mentioned centers for their child’s Vojta therapy, acted as co-therapists and did regular home-based exercises with children, following instructions provided by a certified Vojta therapist according to the child’s functional status.

### 2.2 Participants

The convenience sampling method, not random selection, was used.

The inclusion criteria were established based on the pilot studies: willingness to start their child’s therapy with the Vojta method, conducting the therapy for a minimum period of 2 months children aged 0–1 year, the use of the Vojta method with the child and by the parents for the first time, no previously diagnosed or pharmacologically treated serious mental illnesses (e.g., psychosis, bipolar disorder, depressive disorder). Only one caregiver per family who led the exercises with the child at home was included.

Exclusion criteria were also established: lack of parental consent, the use of other therapeutic methods with the child at the same time, and the application of the Vojta method for less than 2 months.

A total of 111 participants with a mean age of 32.8 years (±4.8) who met the inclusion criteria were recruited for the study and began their child’s therapy (T1). At the final measurement, after 2 months of the child’s therapy (T2), 57 participants with a mean age of 32.8 years (±4.6) were assessed. Only these data (participants who completed both measurement points: T1 and T2) were included in the statistical analysis.

These numbers fall within the ranges of the estimated sample size calculated using the G*Power 3.1 program. Assuming a minimum test power of 0.80 and an effect size of 0.5, the minimum required sample size for repeated measures (Wilcoxon test) was calculated as 23 participants. The required sample size for the analysis of differences between independent groups (Mann–Whitney U test), assuming a test power of 0.80 and an effect size of 0.8, was 54 participants (27 in each group).

The characteristics of the study group are shown in Table 1.

**Table 1:** Characteristics of the study group (*n* = 57).

Parameters	Subgroup	Group, *n* = 57	
		*n*	%
Sex	Women	34	59.6
	Men	23	40.4
Place of residence	Village	8	14
	City > 100,000	49	86
Education	Primary	1	1.8
	Secondary	10	17.5
	High	46	80.7
Economic situation	Employed	52	91.2
	Unemployed	5	8.8
Relationship	Mother	34	59.6
	Father	23	40.4
Physical activity*	Yes	24	42.1
	No	33	57.9

*Physical activity was defined as at least twice a week/min. 150 min.

### 2.3 Procedure

Parents of children with CCD who were qualified for Vojta therapy based on a referral from a neurologist came for their first visit with a certified Vojta therapist. During this first visit, participants were informed about the aim and course of the study and provided written consent to participate. Subsequently, parents independently completed a set of questionnaires in paper form (self-report) in the presence of the therapist (measurement 1—T1). A total of 111 participants took part in the first measurement.

During the visit, parents were instructed on how and how many times to exercise with their child. The frequency of home-based exercises ranged from 2 to 4 sessions per day and was individually adjusted by the therapist according to the child’s functional status.

Therapeutic follow-up visits were conducted by the same physiotherapist, once a week, in order to check the child’s progress, the correctness of performing the exercises and any changes or introduction of new exercises.

In case of any concerns, parents were provided with telephone contact with the therapist to minimize potential worries or technical errors. The selection of therapy/the exercise program was individualized and tailored to the child’s functional needs (Orth et al., 2018; Vojta et al., 2006; Machnia et al., 2026).

After 2 months of therapy, the second measurement (T2) was conducted, during which parents again independently completed the same questionnaires in paper form. A total of 57 participants took part in the second measurement.

The reasons for the withdrawal are unknown. It should be emphasized that this was not a formal withdrawal from participation in the research project, but a failure to attend the subsequent scheduled visits. Preliminary analyses showed that these participants did not differ in terms of sociodemographic characteristics, baseline stress levels or mood compared to the group of 57 participants analysed (Table 2). This is an interesting and surprising phenomenon, but it requires further analysis and research, which, however, goes beyond the scope of this article.

**Table 2:** Comparison between the analyzed group (with T1 and T2; *n* = 57) and group with only T1 (*n* = 54) [Mann–Whitney *U* test and *χ*^2^ test (Fisher’s test)].

Parameters	Group with only T1 *n* = 54		Analysed group with T1 and T2, *n* = 57		*p*	Cohen’s *d*
	Median	IQR	Median	IQR		
Age	32	3	33	5	0.9396	0.02
PSS-10	19.5	10	16	9	0.2686	0.21
PHQ-9	5	6	6	8	0.3625	0.40

Parameters	Subgroup	Group with only T1 *n* = 54		Analysed group with T1 and T2, *n* = 57		*p*	Cramer’s V
		*n*	%	*n*	%		
Sex	Women	35	59.6	34	59.6	0.5748	0.05
	Men	19	40.4	23	40.4		
Education	Primary	3	5.6	1	1.8	0.5862	0.11
	Secondary	8	14.8	10	17.5		
	High	43	79.6	46	80.7		
Economic situation	Employed	50	92.6	52	91.2	1.0000	0.02
	Unemployed	4	7.4	5	8.8		
Relationship	Mother	34	63.0	34	59.6	0.2372	0.15
	Father	20	37.0	23	40.4		
Physical activity	Yes	23	42.1	24	57.9	0.9586	0.01
	No	31	57.9	33	42.1		

T1- measurement 1; T2- measurement 2. PSS-10- perceived stress scale; PHQ-9- patient health questionnaire; IQR – interquartile range. **p < 0.05.

### 2.4 Ethical statement

The parents were informed of the rules and aims of the study and gave their voluntary, written consent to participate. The study was conducted in accordance with the Helsinki Declaration and had approval from the Senate Commission for the Ethics of Scientific Research of the Wroclaw University of Health and Sport Sciences (reference no. 21/2023).

### 2.5 Measurement

The study used the Perceived Stress Scale (PSS-10), the Patient Health Questionnaire (PHQ-9), the Brief COPE Inventory (Mini-COPE) and a self-designed questionnaire.

#### 2.5.1 Perceived stress scale (PSS-10)

The PSS-10 questionnaire consists of 10 items and is used to assess the subjective perception of stress in the context of stressful situations over the past month. This scale is a simple and reliable tool that can be useful in both clinical practice and scientific research. The scale includes six negative items (items 1, 2, 3, 6, 9, and 10), and four positive items (items 4, 5, 7, and 8). Each item in the PSS-10 is rated on a 5-point Likert scale ranging from never (0 points) to very often (4 points). Before calculating the total score, the positively worded items were reverse-coded according to the original scoring procedure. The total score is the sum of responses to all 10 items. The maximum possible score is 40 points. Scores from 0 to 13 indicate low exposure to stress, whereas scores above 20–22 indicate high exposure to stress. The Cronbach’s alpha is 0.86 (Juczyński and Ogińska-Bulik, 2009; Cohen et al., 1983).

#### 2.5.2 Patient health questionnaire (PHQ-9)

The PHQ-9 questionnaire is a screening tool used to assess the severity of depressive symptoms. The Polish version consists of 9 items addressing the presence of depressive symptoms and 1 additional item. Depending on the intensity of a given symptom during the past 2 weeks, respondents select one of the options on a 0–3 scale, where 3 indicates that the symptom occurred most frequently. The maximum total score is 27 points. Cut-off scores of 5, 10, 15, and 20 correspond to mild, moderate, moderately severe, and severe depression, respectively. The PHQ-9 is currently regarded as one of the most reliable instruments for assessing the risk of depression in individuals aged 18 to 60 years. The Cronbach’s alpha is 0.88 (Kokoszka et al., 2016; Kroenke et al., 2001).

#### 2.5.3 Brief COPE inventory (Mini-COPE)

The Mini-COPE inventory, developed by Carver and adapted into Polish by Juczyński and Ogińska-Bulik, is used to assess typical coping strategies and responses in situations of severe stress. It is intended primarily for research purposes, but it can also be applied in clinical practice, screening studies, and preventive interventions to evaluate the effectiveness of therapeutic programs.

The Mini-COPE is a self-report questionnaire consisting of 28 items referring to 14 coping strategies such as active coping, planning, positive reappraisal, acceptance, sense of humor, turning to religion, seeking emotional and instrumental support, self-distraction, denial, venting, substance use, behavioral disengagement, and self-blame. A four-point Likert scale ranging from 0 to 3 is used to rate each item. The score for each strategy is calculated as the sum of the two items assigned to that strategy, then divided by 2. The range of results for each strategy is 0–3 points. Higher scores indicate more frequent use of a given strategy by the respondent. The split-half reliability was 0.86 (Guttman split-half coefficient – 0.87). Strategies such as active coping, planning, and use of instrumental support are referred to as “problem-oriented” and use of positive reappraisal, acceptance, emotional support, religion, or venting are considered “emotion-oriented.” Denial, self-distraction, behavioural disengagement, substance use, self-blame and humour are referred to as avoidance (Juczyński and Ogińska-Bulik, 2009; Carver, 1997).

#### 2.5.4 Self-designed questionnaire

An original questionnaire was used to collect sociodemographic data such as age, parental role (mother/father), place of residence, education and employment status. Additional items concerned sources of knowledge about the Vojta method, concerns related to the therapy and undertaking physical activity. Physical activity was assessed based on self-reported participation (yes/no), duration and frequency of physical activity. It was assumed that being physically active means being active at least twice a week for at least 150 min (according to WHO minimum guidelines). This was the basis for dividing participants into two groups - physically active and inactive - used in the statistical analysis and presented in the Results section.

The above assessments were conducted at the first measurement (T1) and after 2 months of Vojta therapy (T2).

All participating parents were scheduled for a consultation with the same specialist—a certified Vojta method therapist with many years of experience.

### 2.6 Statistical analyses

Given the ordinal nature of most variables and the non-normal distribution of quantitative variables, the median and interquartile range (IQR = Q3–Q1) were used as descriptive statistics. To assess differences between the first (T1) and second measurement (T2), the Wilcoxon signed-rank test for dependent data was applied. Comparisons between groups were performed using the U Mann–Whitney test and *χ*^2^ test (when Cochran’s rule was not satisfied, Fisher’s exact test was used). The level of statistical significance was set at *p* < 0.05. For analyses involving multiple comparisons of the same variable, the Bonferroni correction was applied to control for the cumulative risk of a Type I error. The significance level of *p* = 0.05 was divided by the number of comparisons performed: *p* = 0.05/2 = 0.025.

The effect size for the Mann–Whitney U test was estimated using eta-squared and subsequently transformed into Cohen’s d (Lenhard and Lenhard, 2022). Effect sizes for the *χ*^2^ test and Fisher’s exact test were calculated using Cramér’s V coefficient, whereas the effect size for the Wilcoxon test was calculated using the effect size coefficient r_g_, according to the formula $r_{g}=Z/\sqrt{N}$ (where Z denotes the Wilcoxon test statistic and N the sample size) (Rosnow and Rosenthal, 2003; Prajzner, 2022). All analyses were conducted with Statistica 14.1 and PQStat 1.8.4 software and online statistical calculators: https://www.psychometrica.de/effect_size.html (access 08.06.2026).

## 3 Results

### 3.1 Results in whole group

In the studied group of parents, the most common source of knowledge about the Vojta method was acquaintances (31.6%), followed by the internet (29.8%) and physicians (21.1%). At the same time, the most frequently reported concerns were related to the child’s health (64.9%), followed by the child’s future (33.3%) and the correct performance of the therapy (22.8%). It should be noted that participants were allowed to indicate more than one source of knowledge and more than one concern.

Analysis of the Mini-COPE results showed that, both at measurement 1 (T1) and measurement 2 (T2), the study group of parents most frequently used strategies such as *Active Coping* and *Seeking Emotional Support*. A statistically significant difference was observed between T1 and T2 in strategies such as *Turning to Religion* and *Behavioral Disengagement*. In T2, significantly higher scores were observed in these strategies. Also, a statistically significant improvement in mood (PHQ-9) was noted. Scores for the other coping strategies and parental stress levels (PSS-10) did not differ (Table 3).

**Table 3:** Coping strategies (Mini-COPE) in the total study group (*n* = 57) at T1 and T2 (Wilcoxon test).

Whole group (*n* = 57)	T1		T2		T1 vs. T2	*r_g_*
	Median	IQR	Median	IQR	*p*	
Active coping	2.0*	1.0	2.0*	1.0	0.7390	0.04
Planning	0.5	2.0	0.5	2.0	0.2772	0.14
Positive reframing	1.5	1.0	1.5	1.0	0.3525	0.12
Acceptance	1.5	0.5	1.5	0.5	0.2020	0.17
Sense of Humor	1.0	1.5	1.0	1.0	0.7364	0.04
Turning to religion	0.5	1.0	1.0	1.5	0.0383**	0.27
Seeking emotional Support	2.0*	0.5	2.0*	1.0	0.1329	0.20
Seeking instrumental support	1.5	1.0	1.5	1.0	0.6071	0.07
Self-distraction	1.5	1.0	1.5	1.0	0.1826	0.18
Denial	1.5	2.0	1.0	2.0	0.2452	0.15
Venting	1.0	0.5	1.0	0.5	0.4915	0.09
Psychoactive substance use	1.5	2.0	1.5	2.0	0.9394	0.01
Behavioral disengagement	1.5	1.0	1.5	1.5	0.0261**	0.29
Self-blame	0.5	1.5	0.5	1.0	0.5663	0.08
PSS-10	16.00	9.00	16.00	10.00	0.1018	0.10
PHQ-9	6.00	8.00	4.00	5.00	0.0245**	0.30

T1- measurement 1; T2- measurement 2. PSS-10- perceived stress scale; PHQ-9- patient health questionnaire; IQR – Interquartile Range. *the most commonly employed strategies; **p < 0.05.

### 3.2 Results depending on gender

At T1, women in the study group, compared with men, significantly more often used strategies such as Seeking Emotional Support, Seeking Instrumental Support, and Venting, and demonstrated a higher level of stress (PSS-10). At T2, women, compared with men, significantly more often used the strategies turning to Religion and Venting, and showed significantly worse mood levels (PHQ-9). They still used the strategies Seeking Emotional Support and Seeking Instrumental Support more often, but it was no longer statistically significant after Bonferroni correction (Table 4).

**Table 4:** Coping strategies (Mini-COPE) in the female and male groups at T1 and T2 (Mann–Whitney *U* test).

T1	Women (*n* = 34)		Men (*n* = 23)		*p*	Cohen’s *d*
	Median	IQR	Median	IQR		
Active coping	2.0*	1.0	2.0*	1.0	0.7759	0.08
Planning	0.5	2.0	1.0	2.5	0.3017	0.28
Positive reframing	1.5	1.0	1.5	1.0	0.4894	0.19
Acceptance	1.5	0.5	1.5	1.0	0.4254	0.22
Sense of humor	1.5	1.0	1.0	1.0	0.3093	0.27
Turning to religion	1.0	1.0	0.5	1.0	0.1619	0.38
Seeking emotional support	2.0*	1.0	1.5	1.0	0.0183**	0.66
Seeking instrumental support	1.5	1.0	1.0	0.5	0.0238**	0.63
Self-distraction	1.75	1.0	1.5	1.0	0.5365	0.17
Denial	1.5	2.0	1.0	2.0	0.0847	0.47
Venting	1.5	0.5	1.0	1.0	0.0144**	0.67
Psychoactive substance use	1.5	2.0	1.0	2.0	0.9935	0.01
Behavioral disengagement	1.75	1.0	1.5	1.5	0.2134	0.34
Self-blame	0.75	1.5	0.5	1.0	0.3798	0.24
PSS-10	19.00	8.00	15.00	8.00	0.0270**	0.62
PHQ-9	6.00	8.00	5.00	6.00	0.2668	0.65

T2	Women (*n* = 34)		Men (*n* = 23)		*p*	Cohen’s *d*
	Median	IQR	Median	IQR		
Active coping	2.25*	1.0	2.0*	1.0	0.4543	0.20
Planning	0.5	2.0	0.5	2.0	0.7884	0.07
Positive reframing	1.5	1.0	1.0	0.5	0.2194	0.33
Acceptance	1.5	0.5	1.5	1.0	0.4792	0.19
Sense of humor	1.25	1.5	1.0	1.0	0.2415	0.32
Turning to religion	1.0	1.0	0.5	1.0	0.0223**	0.64
Seeking emotional support	2.0*	1.0	1.5	1.0	0.0481	0.55
Seeking instrumental support	1.5	1.0	1.0	1.0	0.0318	0.60
Self-distraction	1.5	1.0	1.0	1.5	0.0749	0.49
Denial	1.25	1.0	1.0	2.0	0.5802	0.15
Venting	1.25	1.0	1.0	0.5	0.0073**	0.77
Psychoactive substance use	1.5	2.0	1.5	2.0	0.7083	0.10
Behavioral disengagement	1.5	1.0	1.0	1.5	0.0923	0.46
Self-blame	1.0	1.0	0.5	1.0	0.0500	0.54
PSS-10	17.00	9.00	15.00	9.00	0.1570	0.39
PHQ-9	5.50	7.00	3.00	5.00	0.0075**	0.76

T1- measurement 1; T2- measurement 2. PSS-10- perceived stress scale; PHQ-9- patient health questionnaire; IQR – interquartile range; *the most commonly employed strategies. **p < 0.025.

No statistically significant differences in Mini-COPE were observed between T1 and T2 in the male group (*p* > 0.0250); however, a significant improvement in mood was noted (*p* = 0.0117). In contrast, among the female participants, after 2 months of their child’s therapy, a statistically significantly higher score in the strategy Turning to Religion (*p* = 0.0144) was observed, as well as a significant reduction in stress levels (*p* = 0.0206).

### 3.3 Results depending on physical activity

A total of 42% of parents reported regular physical activity. At T1, physically active parents, compared with those who were not physically active, significantly more often used the strategy Active Coping and demonstrated significantly lower stress levels (PSS-10). At T2, physically active parents, compared with inactive parents, significantly more often used the strategy Acceptance and showed significantly better mood levels (PHQ-9) (Table 5).

**Table 5:** Coping strategies (Mini-COPE) in the physically active and inactive groups at T1 and T2 (Mann–Whitney *U* test).

T1	Physically active (*n* = 24)		Physically inactive (*n* = 33)		*p*	Cohen’s *d*
	Median	IQR	Median	IQR		
Active coping	2.5*	1.0	2.0*	1.0	0.0108**	0.78
Planning	1.5	2.5	0.5	2.0	0.4688	0.26
Positive reframing	1.5	1.0	1.5	1.0	0.3553	0.31
Acceptance	1.5	0.5	1.5	1.0	0.3382	0.33
Sense of humor	1.0	1.0	1.0	1.0	0.6771	0.18
Turning to religion	1.0	1.5	0.5	1.0	0.2471	0.38
Seeking emotional support	2.0*	0.5	2.0*	0.5	0.7643	0.15
Seeking instrumental support	1.5	1.0	1.5	1.0	0.5160	0.24
Self-distraction	1.5	1.5	1.5	1.0	0.7707	0.15
Denial	1.5	2.0	1.5	2.0	0.8481	0.12
Venting	1.0	1.0	1.0	0.5	0.3641	0.31
Psychoactive substance use	1.0	2.0	1.5	2.0	0.3957	0.29
Behavioral disengagement	1.5	2.0	1.5	1.0	0.3258	0.33
Self-blame	0.5	1.0	1.0	1.0	0.1027	0.51
PSS-10	14.0	9.0	18.0	7.0	0.0193**	0.72
PHQ-9	5.5	8.0	6.0	7.0	0.7067	0.51

T2	Physically active (*n* = 24)		Physically inactive (*n* = 33)		*p*	Cohen’s *d*
	Median	IQR	Median	IQR		
Active coping	2.5*	0.5	2.0*	1.0	0.3265	0.32
Planning	1.0	2.5	0.5	2.0	0.4992	0.24
Positive reframing	1.5	1.0	1.5	1.0	0.9864	0.08
Acceptance	1.5	0.5	1.5	0.5	0.0244**	0.66
Sense of humor	1.0	1.0	1.0	1.0	0.9594	0.09
Turning to religion	1.0	1.0	0.5	1.0	0.1564	0.44
Seeking emotional support	2.0*	1.0	2.0*	1.0	0.8908	0.11
Seeking instrumental support	1.5	1.0	1.5	1.0	0.4298	0.27
Self-distraction	1.0	1.0	1.5	1.0	0.0682	0.55
Denial	1.0	1.5	1.0	2.0	0.6322	0.19
Venting	1.0	1.0	1.0	0.5	0.6663	0.18
Psychoactive substance use	1.5	2.0	1.0	2.0	0.9247	0.10
Behavioral disengagement	1.0	2.0	1.5	1.0	0.1359	0.46
Self-blame	0.5	1.0	1.0	1.5	0.2833	0.35
PSS-10	14.0	8.0	17.0	9.0	0.0276	0.49
PHQ-9	3.0	4.0	6.0	7.0	0.0125**	0.58

T1- measurement 1; T2- measurement 2. PSS-10- perceived stress scale; PHQ-9- patient health questionnaire; IQR – interquartile range; *the most commonly employed strategies. **p < 0.025.

No statistically significant differences in Mini-COPE were observed between T1 and T2 in the groups of physically active and inactive parents (*p* > 0.025). However, a statistically significant improvement in mood was noted in the physically active group (*p* = 0.0051).

### 3.4 Results depending on stress level

In the total study group at T1, more than 70% (*n* = 40) of parents presented with moderate or high stress levels, while only about 30% had low stress levels. At T1, parents with moderate and high stress, compared with those with low stress, significantly more often used the strategy Venting and Self-blame. After applying the Bonferroni correction, only the strategy Venting remained. At T2, no statistically significant differences were observed in Mini-COPE; however, in the group of parents with moderate and high stress, significantly worse mood and persistently higher stress levels were noted (Table 6).

**Table 6:** Coping strategies (Mini-COPE) at T1 and T2 in the groups with low versus moderate/high stress levels (Mann–Whitney *U* test).

T1	Low stress (*n* = 17)		Moderate/high stress (*n* = 40)		*p*	Cohen’s *d*
	Median	IQR	Median	IQR		
Active coping	2.5*	0.5	2.0*	1.0	0.2425	0.32
Planning	1.0	2.5	0.5	2.0	0.2606	0.30
Positive reframing	1.5	1.0	1.5	1.0	0.9722	0.01
Acceptance	1.5	1.0	1.5	0.8	0.7602	0.08
Sense of humor	1.5	0.5	1.0	1.5	0.8206	0.06
Turning to religion	1.0	1.0	0.5	1.0	0.1164	0.42
Seeking emotional support	1.5	1.0	2.0*	0.5	0.4173	0.22
Seeking instrumental support	1.5	1.0	1.5	1.0	0.7535	0.09
Self-distraction	1.5	1.0	1.5	1.0	0.6883	0.11
Denial	1.5	2.0	1.5	1.5	0.5590	0.16
Venting	0.5	1.0	1.3	0.8	0.0054**	0.80
Psychoactive substance use	1.0	2.0	1.5	2.0	0.4585	0.20
Behavioral disengagement	1.0	2.0	2.0	1.0	0.0528	0.53
Self-blame	0.0	1.0	1.0	1.3	0.0371	0.58
PSS-10	10.0	2.0	20.0	7.0	<0.0001**	2.53
PHQ-9	6.0	8.0	6.0	8.0	0.5748	0.49

T2	Low stress (*n* = 17)		Moderate/high stress (*n* = 40)		*p*	Cohen’s *d*
	Median	IQR	Median	IQR		
Active coping	2.0*	1.0	2.0*	1.0	0.7802	0.08
Planning	1.5	2.5	0.5	2.0	0.5074	0.18
Positive reframing	1.5	1.0	1.5	1.0	0.5358	0.17
Acceptance	1.5	0.5	1.5	0.5	0.9652	0.01
Sense of humor	1.0	0.5	1.0	1.5	0.7469	0.09
Turning to religion	1.0	1.0	1.0	1.0	0.1577	0.38
Seeking emotional support	1.5	0.5	2.0*	1.0	0.2953	0.28
Seeking instrumental support	1.5	1.0	1.5	1.0	0.9652	0.01
Self-distraction	1.5	1.5	1.5	1.0	0.6130	0.14
Denial	1.5	2.0	1.0	1.8	0.7668	0.08
Venting	1.0	0.5	1.0	0.5	0.0487	0.54
Psychoactive substance use	1.0	1.5	1.5	2.0	0.6819	0.11
Behavioral disengagement	1.0	1.0	1.5	1.0	0.0907	0.46
Self-blame	0.5	1.0	1.0	1.5	0.0890	0.46
PSS-10	11.0	10.0	17.5	7.0	0.0023**	0.88
PHQ-9	3.0	3.0	6.5	6.0	0.0018**	0.90

T1- measurement 1; T2- measurement 2. PSS-10- perceived stress scale; PHQ-9- patient health questionnaire; IQR – interquartile range. *the most commonly employed strategies; **p < 0.025.

No statistically significant differences (*p* > 0.0250) in Mini-COPE, PSS-10 and PHQ-9 were observed between T1 and T2 in either group.

### 3.5 Results depending on depression symptoms

In the total study group at T1, more than 56% (*n* = 32) of parents showed suspected depression (PHQ-9 > 5). At T1, parents with depression significantly more often used the strategy Seeking Instrumental Support and Positive Reframing (after the Bonferroni correction, only Seeking Instrumental Support) compared with parents without depression. At T2, parents with depression still significantly more often used the strategies Seeking Instrumental Support and Positive Reframing (Table 7).

**Table 7:** Coping strategies (Mini-COPE) in the groups with and without depression at T1 (Mann–Whitney U test).

T1	Without depression (*n* = 25)		With depression (*n* = 32)		*p*	Cohen’s *d*
	Median	IQR	Median	IQR		
Active coping	2.0*	0.5	2.0*	1.0	0.6995	0.11
Planning	0.0	1.5	1.0	2.3	0.0680	0.50
Positive reframing	1.0	0.5	1.5	1.0	0.0479	0.55
Acceptance	1.5	0.5	1.5	1.0	0.8722	0.05
Sense of humor	1.5	1.0	1.0	1.0	0.2777	0.29
Turning to religion	1.0	0.5	0.5	1.0	0.6352	0.13
Seeking emotional support	2.0*	0.5	2.0*	0.8	0.2959	0.28
Seeking instrumental support	1.0	0.5	1.8	0.8	0.0080**	0.75
Self-distraction	1.5	1.5	1.8	1.0	0.1642	0.38
Denial	1.5	2.0	1.5	1.8	0.5253	0.17
Venting	1.0	1.0	1.5	0.5	0.1226	0.42
Psychoactive substance use	1.5	1.5	1.5	2.0	0.7538	0.09
Behavioral disengagement	1.5	0.5	1.3	1.5	0.5681	0.15
Self-blame	0.5	1.0	0.8	1.5	0.3678	0.24
PSS-10	16.0	10.0	15.5	9.5	0.6995	0.11
PHQ-9	2.5	3.0	9.0	6.0	<0.0001**	3.26

T2	Without depression (*n* = 25)		With depression (*n* = 32)		p	Cohen’s *d*
	Median	IQR	Median	IQR		
Active coping	2.0*	1.0	2.0*	1.0	0.7969	0.07
Planning	0.5	1.5	1.3	2.3	0.0517	0.54
Positive reframing	1.0	0.5	1.5	1.0	0.0224**	0.64
Acceptance	1.5	0.0	1.5	1.0	0.8596	0.05
Sense of humor	1.5	1.0	1.0	1.0	0.2672	0.30
Turning to religion	1.0	1.0	1.0	1.5	0.9679	0.01
Seeking emotional support	2.0*	0.5	2.0*	1.0	0.1716	0.37
Seeking instrumental support	1.0	0.5	1.5	1.0	0.0076**	0.76
Self-distraction	1.0	1.5	1.5	1.0	0.3427	0.26
Denial	1.0	1.5	1.0	1.8	0.7175	0.10
Venting	1.0	1.0	1.0	0.5	0.5790	0.15
Psychoactive substance use	1.5	2.0	1.0	2.0	0.7907	0.07
Behavioral disengagement	1.5	1.0	1.0	1.5	0.7599	0.08
Self-blame	0.5	1.0	1.0	1.5	0.2436	0.32
PSS-10	17.0	11.0	16.5	8.0	0.8156	0.06
PHQ-9	4.0	6.0	4.0	6.0	0.3536	0.25

T1- measurement 1; T2- measurement 2. PSS-10- perceived stress scale; PHQ-9- patient health questionnaire; IQR – interquartile range. *the most commonly employed strategies; **p < 0.025.

No statistically significant differences in Mini-COPE were observed between T1 and T2 in the studied groups (*p* > 0.0250). However, a significant improvement in mood (*p* = 0.0003) was noted in the group of parents with depression.

## 4 Discussion

The aim of the present study was to conduct a detailed analysis of stress-coping strategies used by parents of children with CCD undergoing Vojta therapy in the context of their perceived stress levels and mood over a period of 2 months.

Research on parental stress and coping strategies is important, as it contributes to a better understanding of the factors associated with this phenomenon and may support the development of appropriate supportive interventions (Howe et al., 2014).

Our findings indicate that at both T1 and T2, the studied group of parents most frequently used Active Coping and Seeking Emotional Support, strategies commonly described as more adaptive because they facilitate active problem solving and mobilization of social resources. These findings may be interpreted within the transactional model of stress and coping proposed by Lazarus and Folkman, according to which individuals attempt to manage stressful demands through both problem-focused and emotion-focused coping strategies. The frequent use of Active Coping may reflect efforts to maintain a sense of control over the child’s rehabilitation process, whereas Seeking Emotional Support may indicate the need to regulate the emotional burden associated with long-term caregiving and the role of an active co-therapist. The obtained results also indicate that parents do not predominantly rely on a single category of coping strategies, but rather combine problem-focused and emotion-focused strategies, which is consistent with previous research demonstrating flexible use of different strategies depending on the situation (Folkman and Lazarus, 1985; Leslie-Miller et al., 2024). However, the lack of a control group limits the interpretation of whether the observed coping patterns were specific to Vojta therapy or reflected more general parental adaptation processes. Statistically significant differences between T1 and T2 were observed in the strategies turning to Religion and Behavioral Disengagement, as well as improvement in mood. Previous studies involving parents of children with chronic illnesses or disabilities have examined coping strategies as potential moderators of the relationship between caregiving stress and quality of life (Dardas and Ahmad, 2015), as well as between the complexity of care and parental stress levels (Lyons et al., 2010). These findings may be considered in relation to our results, as they also point to the possible role of coping strategies, such as active coping and seeking social support, in the context of parental stress. Previous studies indicate that coping strategies based on seeking social support as well as avoidance/escape may serve a buffering function in the relationship between parental stress and quality of life (Lyons et al., 2010), whereas more frequent use of problem-focused strategies and less reliance on emotion-focused strategies are associated with lower levels of depressive symptoms among mothers of adolescents with developmental disorders (Woodman and Hauser-Cram, 2013).

The results of the present study indicate that at T1, women significantly more often used the emotional-oriented strategies such as Seeking Emotional Support, Seeking Instrumental Support, and Venting, and their stress levels were higher than those of men. At T2, women continued to employ the Venting strategy and more frequently engaged in Turning to Religion, which was accompanied by poorer mood. The study by Byra and Parchomiuk showed that mothers coped with difficulties through acceptance, problem-solving, and also seeking emotional support (Byra and Parchomiuk, 2018). Strojek et al. found that mothers significantly more often chose strategies of seeking instrumental support and turning to religion, which was also confirmed by the observations of Aftyka et al., who emphasized that women do not limit themselves to a single group of strategies, but significantly more often than men choose instrumental support seeking (Strojek et al., 2022; Aftyka et al., 2015). Although the literature points to a tendency to gradually reduce less adaptive strategies over time (Vernhet et al., 2022; Li et al., 2012), our observations suggest that this process may require a longer period of monitoring, because a two-month follow-up period may be insufficient to detect meaningful changes in coping strategies, which are often considered relatively stable psychological constructs. This may indicate the need for further research to capture the dynamics of changes in coping strategies used by mothers and fathers caring for a child with CCD. No significant differences in coping strategies between T1 and T2 were observed in men, although their mood improved, whereas among women, after 2 months of the child’s therapy, a significant increase in the use of the Turning to Religion strategy was recorded, along with a reduction in stress levels. These findings may be considered in the context of previous reports. These results somewhat differ from reports indicating that parents of children with DD are less likely to resort to strategies involving seeking emotional support in religion compared to parents of typically developing children (An and Palisano, 2014). At the same time, they may also be viewed in relation to other studies emphasizing the beneficial effect of religious support (Isa et al., 2017), which highlights the ambiguous nature of previous findings in this area (Amireh, 2019; Beighton and Wills, 2017). These discrepancies may result from differences in study populations, cultural backgrounds, or the specific caregiving demands associated with particular developmental conditions and therapeutic approaches. Despite the more frequent and more diverse use of coping strategies by mothers, this does not translate into better emotional functioning. On the contrary, women continue to exhibit higher levels of stress and worse mood compared to fathers, which may indicate that the intensity or diversity of coping strategies alone is insufficient for effective regulation of psychological burden. Greater emotional involvement may also contribute to heightened vigilance and stronger emotional responses to the child’s discomfort during therapy. Therefore, these findings highlight the need for targeted psychological support for mothers of children with CCD.

Regular physical activity was associated with reducing the risk of depression, alleviating its symptoms, supporting the process of treatment and recovery, lowering the risk of relapse, and reducing the psychological burden on caregivers of individuals struggling with this disorder (Babyak et al., 2000; Motl et al., 2004; Abu-Omar et al., 2004; Hoffman et al., 2011; Christofoletti et al., 2011; Schuch et al., 2016; Kvam et al., 2016). The results obtained in our study are consistent with these reports and may suggest that physically active parents demonstrated better stress management, a reduction in depressive symptoms, and the prevention of mood disorders (Babyak et al., 2000; Motl et al., 2004; Hoffman et al., 2011; Schuch et al., 2018; Kandola et al., 2018). Previous studies have indicated that regular exercise promotes mood improvement, decreases stress levels, and lowers the risk of recurrent depressive episodes (Schuch et al., 2018; Kandola et al., 2018; Goodarzi et al., 2024). Our study complements these observations by showing that physically active parents demonstrated not only better mood and lower perceived stress, but also more frequent use of adaptive coping strategies, such as Active Coping and Acceptance. Importantly, no statistically significant differences were found in the coping strategies employed between T1 and T2 in the groups of physically active and inactive parents. At the same time, a significant improvement in mood was observed among physically active parents, suggesting a potential association between physical activity and psychological well-being in this population.

It is worth noting that the results of pilot studies indicate that physically inactive parents most often chose the coping strategy referred to as Behavioral Disengagement, meaning that they were much more likely than physically active individuals to give up on striving toward goal attainment. This strategy is ineffective and may provide only short-term relief (Folkman and Lazarus, 1985). Physically inactive parents were also characterized by higher stress levels, and according to the findings of Repka et al., the higher the levels of stress and fatigue, the more frequently the Behavioral Disengagement strategy was employed (Repka et al., 2019). However, in the present study, no statistically significant differences were observed between the groups in terms of coping strategies used, indicating that these observations should be interpreted with caution and physical activity may be more strongly associated with psychological well-being than with the specific coping strategies employed by parents of children undergoing intensive rehabilitation.

At T1, more than 70% of parents presented with moderate or high levels of stress, and individuals in this group significantly more often used the Venting strategy compared to parents with low stress levels. In the second measurement (T2), no significant differences were observed in the strategies employed; however, parents with higher stress levels continued to exhibit poorer mood and higher stress which may be considered in relation to findings indicating that differences in psychological well-being among parents of children with DD may persist over time (Neece et al., 2012), potentially due to the chronic nature of caregiving-related stress (Sloan et al., 2020). The relatively stable stress levels observed over the two-month period may reflect the process of ongoing cognitive appraisal described in the transactional model of stress and coping. According to this model, stress depends not only on objective demands but also on how individuals evaluate their available resources to cope with those demands. Parents may gradually adapt to the therapeutic demands without perceiving a substantial reduction in the objective burden associated with intensive caregiving and home-based rehabilitation. These findings are consistent with the observations of Antonopoulou et al., who did not identify significant differences in coping strategies between groups of parents over a longer time perspective, but pointed to the adverse impact of emotion-focused strategies on psychological well-being (Antonopoulou et al., 2020). These findings may suggest that, in this group of parents, coping strategies based on emotional regulation, such as Venting, may not lead to significant improvements in psychological well-being. This may indicate that, in the context of chronic caregiving stress, interventions should be oriented toward more effective coping strategies that go beyond spontaneous emotional responses (Neece et al., 2012).

More than half of the parents at T1 presented with symptoms of depression (PHQ-9 > 5), and individuals in this group more frequently used strategies of Seeking Instrumental Support and, at T2, also Positive Reframing. Although the comparative analysis did not reveal significant changes in coping strategies, a significant improvement in mood was observed in the group of parents with depression. They are partially consistent with previous research (Meleady et al., 2020) and suggest that even with stable coping patterns, an improvement in psychological well-being is possible, highlighting the importance of support accompanying the child’s rehabilitation process. This pattern may be explained by both compensatory coping mechanisms and measurement-related factors. On the one hand, parents experiencing higher levels of stress may be more likely to engage in cognitively oriented coping strategies, such as positive reappraisal, in order to regulate their emotional state. Previous research also suggests that coping strategies may co-occur with high levels of stress and do not necessarily reflect lower psychological burden (Folkman and Moskowitz, 2000). On the other hand, it should be taken into account that the assessment of coping strategies based on self-report questionnaires may be susceptible to distortions resulting from social desirability bias, which involves a tendency to present oneself in a more favorable light (van de Mortel, 2008).

From a clinical perspective, the observed changes between T1 and T2 appear to be of moderate relevance. Although no significant changes in coping strategies were identified, improvements in mood and reductions in stress levels in selected groups (e.g., women, physically active parents, and parents with depressive symptoms) suggest that psychological well-being may improve even without substantial modifications in coping patterns. However, due to the absence of a control group, these findings and their clinical implications should be interpreted with caution, as the observed psychological changes cannot be unequivocally attributed to participation in the rehabilitation process itself. Future studies should include longer follow-up periods, larger study groups, and control groups in order to better evaluate long-term changes in coping strategies and psychological functioning among parents of children undergoing intensive rehabilitation.

The obtained results indicate that, in the studied group of parents, higher levels of stress are consistently associated with poorer mood and tend to persist over time, whereas improvements in psychological well-being may occur independently of significant changes in the coping strategies used. From a clinical perspective, these findings underscore the importance of regular assessment of parental stress and mood, as persistently high stress levels may adversely affect psychological functioning, whereas improvements in well-being may occur without corresponding changes in the coping strategies used.

The role of parents as active co-therapists may create a unique form of psychological burden, combining emotional caregiving responsibilities with pressure related to the effectiveness and regularity of therapeutic exercises performed at home. Therefore, planned interventions for parents of children with CCD undergoing rehabilitation with the Vojta method could be considered to focus on strengthening personal resources by fostering adaptive coping strategies, particularly problem-oriented coping strategies to improve their psychological well-being. This holds important consequences for interdisciplinary approaches within pediatric rehabilitation practices and potential implications for family-centered rehabilitation programs.

### 4.1 Limitations

The conducted study, using selected standardized questionnaires, had a screening character and did not constitute a diagnostic assessment. The study also did not take into account the severity of disorders in children, the lack of detailed information regarding the clinical status of the children, including the severity of motor difficulties, developmental status, comorbidities, and baseline functional impairments. These factors may influence parental stress levels and coping strategies and therefore could have affected the observed results. However, medical records indicated that the condition of children who continued the two-month therapy improved compared with the initial assessment. Future studies should include a more comprehensive characterization of children’s clinical profiles.

Another limitation of this study was the high dropout rate between the first and second measurements (out of 111 participants, only 57 completed the study). The reasons for withdrawal are unknown. It is important to emphasize that this was not a withdrawal from participation in the research project itself; rather, the parents simply did not attend the subsequent scheduled visits. The analyzed strategies can also be related. It is possible that the requirement to take on the role of a therapist—demanding regularity and work with one’s own often crying child—may have led some parents to choose alternative therapeutic methods. This is an interesting and surprising phenomenon that we highlight but requires further analysis and research, which, however, goes beyond the scope of this article.

Due to the large number of items and the time-consuming nature of filling out questionnaires regarding stress, mood, coping strategies, and also in situations requiring care of a restless and crying child, it was decided to use survey questions to assess physical activity. Therefore, physical activity was assessed using self-reported questionnaire data, including information on frequency and duration, without objective measures.

It should also be noted that the two-month follow-up period may be insufficient to capture meaningful and sustained changes in coping strategies and stress levels among parents. Coping strategies are dynamic in nature and may require a longer period to show stable changes. This limitation is particularly important given the relatively small longitudinal changes observed in coping strategies during the study period. Therefore, future studies should include a longer follow-up period to allow for a more comprehensive assessment of the effects of therapy over time. The obtained study results encourage their continuation, but with a control group and a larger group of parents.

## 5 Conclusion

The results of the study suggested that parents of children with CCD most frequently used strategies such as Active Coping and Seeking Emotional/ Instrumental Support. At the same time, individuals with higher levels of stress and poorer mood more frequently used strategies such as Venting and Behavioral Disengagement.

Female participants more often chose emotional and religious strategies than males. Moreover, parents with depressive symptoms more frequently used strategies such as Seeking Instrumental Support and Positive Reframing.

The results demonstrate associations between physical activity, lower stress levels, and better mood. The observational design does not permit causal conclusions. Perhaps regular physical activity undertaken by parents of children with CCD may be an important factor in coping with stress, as well as in the conscious and purposeful selection of coping strategies to maintain psychological well-being. However, this requires further research and analysis.

Providing parents with guidance on beneficial coping strategies and encouraging them to engage in physical activity through brief psychological counseling may improve their quality of life and enhance the effectiveness of care for their ill child. Additional attention should be given to mothers, who may experience greater emotional burden related to everyday therapeutic responsibilities. However, due to the absence of a control group, it remains unclear whether the observed coping patterns were specific to parents involved in Vojta therapy or reflected more general experiences associated with parenting a child with symptoms of developmental disorders.

## Data Availability

The original contributions presented in the study are included in the article/supplementary material, further inquiries can be directed to the corresponding author.

## References

[ref1] Abu-Omar K. Rütten A. Lehtinen V. (2004). Mental health and physical activity in the European Union. Soz. Praventivmed. 49, 301–309. doi: 10.1007/s00038-004-3109-8, 15497649

[ref3] Aftyka A. Kopeć A. Wróbel A. Rozalska-Walaszek I. Rzońca P. Rybojad B. . (2015). Experiences of parents of children hospitalized in neonatal intensive care unit: coping strategies, perceived stress and symptoms of posttraumatic stress disorder. Curr. Probl. Psychiatry. 16, 75–80.

[ref4] Allen K. A. Bowles T. V. Weber L. L. (2013). Mothers and fathers stress associated with parenting a child with autism spectrum disorder. Autism Insigh. 5, 1–11.

[ref5] Alós F. J. García A. G. Maldonado M. A. (2022). Coping strategies in parents of children with disabilities: a case-control study. Brain Behav. 12:e2701. doi: 10.1002/brb3.2701, 35833635 PMC9392513

[ref6] Amireh M. M. H. (2019). Stress levels and coping strategies among parents of children with autism and Down syndrome: the effect of demographic variables on levels of stress. Child Care Pract. 25, 146–156. doi: 10.1080/13575279.2018.1446907

[ref7] An M. Palisano R. J. (2014). Family-professional collaboration in pediatric rehabilitation: a practice model. Disabil. Rehabil. 36, 434–440. doi: 10.3109/09638288.2013.797510, 23713990

[ref8] Antonopoulou K. Manta N. Maridaki-Kassotaki K. Kouvava S. Stampoltzis A. (2020). Parenting and coping strategies among parents of children with and without autism: the role of anxiety and emotional expressiveness in the family. Austin J. Autism Relat. Disabil. 6:1054.

[ref9] Aratti A. Zampini L. (2024). Caregiver burden, parenting stress and coping strategies: the experience of parents of children and adolescents with osteogenesis imperfecta. Healthcare (Basel) 12:1018. doi: 10.3390/healthcare12101018, 38786428 PMC11121070

[ref10] Babyak M. Blumenthal J. A. Herman S. Khatri P. Doraiswamy M. Moore K. . (2000). Exercise treatment for major depression: maintenance of therapeutic benefit at 10 months. Psychosom. Med. 62, 633–638. doi: 10.1097/00006842-200009000-00006, 11020092

[ref11] Banaszek G. (2010). Vojta’s method as the early neurodevelopmental diagnosis and therapy concept. Przegl. Lek. 67, 67–76. 20509579

[ref12] Barroso N. E. Mendez L. Graziano P. A. Bagner D. M. (2018). Parenting stress through the lens of different clinical groups: a systematic review & meta-analysis. J. Abnorm. Child Psychol. 46, 449–461. doi: 10.1007/s10802-017-0313-6, 28555335 PMC5725271

[ref13] Bawalsah J. A. (2016). Stress and coping strategies in parents of children with physical, mental, and hearing disabilities in Jordan. Int. J. Educ. 8, 1–22. doi: 10.5296/ije.v8i1.8811

[ref14] Beighton C. Wills J. (2017). Are parents identifying positive aspects to parenting their child with an intellectual disability or are they just coping? A qualitative exploration. J. Intellect. Disabil. 21, 325–345. doi: 10.1177/1744629516656073, 27352854 PMC5703033

[ref15] Blank R Smits-Engelsman B Polatajko H Wilson P; European academy for childhood disability. European academy for childhood disability (EACD): recommendations on the definition, diagnosis and intervention of developmental coordination disorder (long version). Dev. Med. Child Neurol. (2012) 54:54–93. doi:10.1111/j.1469-8749.2011.04171.x, 22171930

[ref16] Bujnowska A. M. Rodriguez C. Garcia T. Areces D. Marsh N. V. (2019). Parenting and future anxiety: the impact of having a child with developmental disabilities. Int. J. Environ. Res. Public Health 16:668. doi: 10.3390/ijerph16040668, 30823540 PMC6406654

[ref17] Byra S. Parchomiuk M. (2018). Resilience a strategie radzenia sobie z problemami u matek dzieci z niepełnosprawnością. Niepełnosprawność 31, 24–41. doi: 10.4467/25439561.NP.18.040.10440

[ref18] Carlson J. M. Miller P. A. (2017). Family burden, child disability, and the adjustment of mothers caring for children with epilepsy: role of social support and coping. Epilepsy Behav. 68, 168–173. doi: 10.1016/j.yebeh.2017.01.013, 28199920

[ref19] Carver C. S. (1997). You want to measure coping but your protocol’s too long: consider the brief COPE. Int. J. Behav. Med. 4, 92–100. doi: 10.1207/s15327558ijbm0401_6, 16250744

[ref20] Castilla A. Gonzalez M. Kysh L. Pico D. Kaplan S. L. Sargent B. (2023). Informing the physical therapy management of congenital muscular torticollis clinical practice guideline: a systematic review. Pediatr. Phys. Ther. 35, 190–200. doi: 10.1097/PEP.0000000000000993, 36637442

[ref21] Christofoletti G. Oliani M. M. Bucken-Gobbi L. T. Gobbi S. Beinotti F. Stella F. (2011). Physical activity attenuates neuropsychiatric disturbances and caregiver burden in patients with dementia. Clinics 66, 613–618. doi: 10.1590/S1807-59322011000400015, 21655755 PMC3093791

[ref22] Cohen S. Kamarck T. Mermelstein R. (1983). A global measure of perceived stress. J. Health Soc. Behav. 24, 385–396. doi: 10.2307/2136404, 6668417

[ref23] Craig F. Savino R. Fanizza I. Lucarelli E. Russo L. Trabacca A. (2020). A systematic review of coping strategies in parents of children with attention deficit hyperactivity disorder (ADHD). Res. Dev. Disabil. 98:103571. doi: 10.1016/j.ridd.2020.103571, 31931455

[ref24] Cuzzocrea F. Murdaca A. M. Costa S. Filippello P. Larcan R. (2016). Parental stress, coping strategies and social support in families of children with a disability. Child Care Pract. 22, 3–19. doi: 10.1080/13575279.2015.1064357

[ref25] Czajkowska M. Kaptur E. (2016). Metoda Vojty w diagnozie i terapii triady ssanie–połykanie–oddychanie u dziecka przedwcześnie urodzonego. Logopedia 45, 205–219.

[ref26] Dardas L. A. Ahmad M. M. (2015). Coping strategies as mediators and moderators between stress and quality of life among parents of children with autistic disorder. Stress. Health 31, 5–12. doi: 10.1002/smi.2513, 23868562

[ref27] Davidson G. Bunting L. McCartan C. Grant A. McBride O. Mulholland C. . (2024). Parental physical activity, parental mental health, children’s physical activity, and children’s mental health. Front. Psych. 15:1405783. doi: 10.3389/fpsyt.2024.1405783, 39015881 PMC11250656

[ref28] Dytrych G. (2008). Controversies concerning Vojta method – in physiotherapists’ opinion. Neurol. Dziec. 17, 59–62.

[ref29] Einfeld S. Emerson E. (2008). “Intellectual disability,” in Rutter’s Child and Adolescent Psychiatry, eds. Rutter M. Bishop D. V. M. Pine D. S. Scott S. Stevenson J. Thapar A. (Hoboken, NJ: John Wiley & Sons Inc.).

[ref30] Endler N. S. Parker J. D. (1990). Multidimensional assessment of coping: a critical evaluation. J. Pers. Soc. Psychol. 58, 844–854. doi: 10.1037/0022-3514.58.5.844 2348372

[ref31] Fang Y. Luo J. Boele M. Windhorst D. van Grieken A. Raat H. (2022). Parent, child, and situational factors associated with parenting stress: a systematic review. Eur. Child Adolesc. Psychiatry 33, 1687–1705. doi: 10.1007/s00787-022-02027-1, 35876894 PMC11211171

[ref32] Folkman S. Lazarus R. S. (1985). If it changes it must be a process: study of emotion and coping during three stages of a college examination. J. Pers. Soc. Psychol. 48, 150–170. doi: 10.1037/0022-3514.48.1.150, 2980281

[ref33] Folkman S. Moskowitz J. T. (2000). Positive affect and the other side of coping. Am. Psychol. 55, 647–654. doi: 10.1037/0003-066X.55.6.647, 10892207

[ref34] Frohlich A. (1998). Stymulacja od Podstaw. Warszawa: WSiP.

[ref35] García-Bravo C. Martínez-Piédrola R. (2023). Living with and managing seizures among parents of children diagnosed with Phelan-McDermid syndrome: a qualitative study using in-depth interviews. Eur. J. Pediatr. 183, 253–262. doi: 10.1007/s00431-023-05285-6, 37870610

[ref36] Goodarzi S. Teymouri Athar M. M. Beiky M. Fathi H. Nakhaee Z. Parvizi Omran S. . (2024). Effect of physical activity for reducing anxiety symptoms in older adults: a meta-analysis of randomized controlled trials. BMC Sports Sci. Med. Rehabil. 16:153. doi: 10.1186/s13102-024-00947-w, 39014515 PMC11251295

[ref37] Gordon A. M. (2011). To constrain or not to constrain, and other stories of intensive upper extremity training for children with unilateral cerebral palsy. Dev. Med. Child Neurol. 53, 56–61. doi: 10.1111/j.1469-8749.2011.04066.x, 21950396

[ref38] Halstead E. J. Griffith G. M. Hastings R. P. (2018). Social support, coping, and positive perceptions as potential protective factors for the well-being of mothers of children with intellectual and developmental disabilities. Int. J. Dev. Disabil. 64, 288–296. doi: 10.1080/20473869.2017.1329192, 34141317 PMC8115529

[ref2] Haripriya A. Deepthi D. P. (2023). Parental self-efficacy and proactive coping among parents of children with neurodevelopmental disorders and parents of typically developed children. Int. J. Indian Psychol. 11, 2086–2097. doi: 10.25215/1103.195

[ref39] Hayes S. A. Watson S. L. (2013). The impact of parenting stress: a meta-analysis of studies comparing the experience of parenting stress in parents of children with and without autism spectrum disorder. J. Autism Dev. Disord. 43, 629–642. doi: 10.1007/s10803-012-1604-y, 22790429

[ref40] Hoffman B. M. Babyak M. A. Craighead W. E. Sherwood A. Doraiswamy P. M. Coons M. J. . (2011). Exercise and pharmacotherapy in patients with major depression: one-year follow-up of the SMILE study. Psychosom. Med. 73, 127–133. doi: 10.1097/PSY.0b013e31820433a5, 21148807 PMC3671874

[ref41] Hou Y. M. Stewart L. Iao L. S. Wu C. C. (2018). Parenting stress and depressive symptoms in Taiwanese mothers of young children with autism spectrum disorder: association with children’s behavioural problems. J. Appl. Res. Intellect. Disabil. 31, 1113–1121. doi: 10.1111/jar.12471, 29790634

[ref42] Howe T. H. Sheu C. F. Wang T. N. Hsu Y. W. (2014). Parenting stress in families with very low birth weight preterm infants in early infancy. Res. Dev. Disabil. 35, 1748–1756. doi: 10.1016/j.ridd.2014.02.015, 24656293

[ref43] Huang X. Zhang H. Chen S. (2019). Neuropsychiatric symptoms, parenting stress and social support in Chinese mothers of children with autism spectrum disorder. Curr Med Sci. 39, 291–297. doi: 10.1007/s11596-019-2033-3, 31016524

[ref44] Isa S. N. I. Ishak I. Ab Rahman A. Saat N. M. Din N. C. Lubis S. H. . (2017). Perceived stress and coping styles among Malay caregivers of children with learning disabilities in Kelantan. Malays. J. Med. Sci. 24, 81–93. doi: 10.21315/mjms2017.24.1.9, 28381931 PMC5346006

[ref45] Jean Y. Q. Mazlan R. Ahmad M. Maamor N. (2018). Parenting stress and maternal coherence: mothers with deaf or hard-of-hearing children. Am. J. Audiol. 27, 260–271. doi: 10.1044/2018_AJA-17-0093, 30007031

[ref46] Juczyński Z. Ogińska-Bulik N. (2009). Narzędzia Pomiaru Stresu i Radzenia Sobie ze Stresem. Warszawa: Pracownia Testów Psychologicznych.

[ref47] Jung M. W. Landenberger M. Jung T. Lindenthal T. Philippi H. (2017). Vojta therapy and neurodevelopmental treatment in children with infantile postural asymmetry: a randomized controlled trial. J. Phys. Ther. Sci. 29, 301–306. doi: 10.1589/jpts.29.301, 28265162 PMC5332993

[ref48] Kandola A. Vancampfort D. Herring M. Rebar A. Hallgren M. Firth J. . (2018). Moving to beat anxiety: epidemiology and therapeutic issues with physical activity for anxiety. Curr. Psychiatry Rep. 20:63. doi: 10.1007/s11920-018-0923-x, 30043270 PMC6061211

[ref49] Karaivazoglou K. Papadaki E. Iconomou G. Touliatos G. Kotsopoulos S. Assimakopoulos K. (2018). Psychological distress and health-related quality of life in parents of children referred to an outpatient service for children with developmental disorders. Australas. Psychiatry 27, 152–156. doi: 10.1177/1039856218815754, 30474387

[ref50] Khavanskaya H. N. Niedźwiecka E. Shpakou A. I. (2020). Parents’ knowledge about stimulation of psychomotor development of premature infant. Wspol. Probl. Hig. Prom. Med. Srod. 10, 247–259.

[ref51] Kokoszka A. Jastrzębski A. Obrębski M. (2016). Psychometric properties of the polish version of patient health Questionnaire-9. Psychiatria 13, 187–193.

[ref52] Kroenke K. Spitzer R. L. Williams J. B. (2001). The PHQ-9: validity of a brief depression severity measure. J. Gen. Intern. Med. 16, 606–613. doi: 10.1046/j.1525-1497.2001.016009606.x, 11556941 PMC1495268

[ref53] Kvam S. Kleppe C. L. Nordhus I. H. Hovland A. (2016). Exercise as a treatment for depression: a meta-analysis. J. Affect. Disord. 202, 67–86. doi: 10.1016/j.jad.2016.03.063, 27253219

[ref54] Lenhard W. Lenhard A. (2022). Computation of effect sizes. Available from: https://www.psychometrica.de/effect_size.html (accessed June 8, 2026).

[ref55] Leslie-Miller C. J. Joormann J. Quinn M. E. (2024). Coping flexibility: match between coping strategy and perceived stressor controllability predicts depressed mood. Affect. Sci. 6, 94–103. doi: 10.1007/s42761-024-00275-9, 40094042 PMC11903985

[ref56] Li R. Cooper C. Bradley J. Shulman A. Livingston G. (2012). Coping strategies and psychological morbidity in family carers of people with dementia: a systematic review and meta-analysis. J. Affect. Disord. 139, 1–11. doi: 10.1016/j.jad.2011.05.055, 21723617

[ref57] Lyons A. M. Leon S. C. Roecker Phelps C. E. Dunleavy A. M. (2010). The impact of child symptom severity on stress among parents of children with ASD: the moderating role of coping styles. J. Child Fam. Stud. 19, 516–524. doi: 10.1007/s10826-009-9323-5

[ref58] Machnia M. Płusajski A. Leśniak E. Urazińska K. Kałużyński W. (2026). Comparison of the effectiveness of Vojta therapy and the NDT Bobath concept in the treatment of congenital muscular torticollis in infants—a retrospective cohort pilot study. J. Clin. Med. 15:1286. doi: 10.3390/jcm15031286, 41682966 PMC12897582

[ref59] McConnell D. Parakkal M. Savage A. Rempel G. (2015). Parent-mediated intervention: adherence and adverse effects. Disabil. Rehabil. 37, 864–872. doi: 10.3109/09638288.2014.946157, 25073583

[ref60] Medina-Mirapeix F. Lillo-Navarro C. (2017). Predictors of parents’ adherence to home exercise programs for children with developmental disabilities, regarding both exercise frequency and duration: a survey design. Eur. J. Phys. Rehabil. Med. 53:545. doi: 10.23736/S1973-9087.17.04464-1, 28466627

[ref61] Medina-Mora M. E. Robles R. Rebello T. J. Domínguez T. Martínez N. Juárez F. . (2019). ICD-11 guidelines for psychotic, mood, anxiety and stress-related disorders in Mexico: clinical utility and reliability. Int. J. Clin. Health Psychol. 19, 1–11. doi: 10.1016/j.ijchp.2018.09.003, 30619492 PMC6300716

[ref62] Meleady J. Nearchou F. Bramham J. Carr A. (2020). Family adaptation among parents of children on the autism spectrum without a comorbid intellectual disability: a test of the double ABCX model. Res. Autism Spectr. Disord. 78:101637. doi: 10.1016/j.rasd.2020.101637

[ref63] Motl R. W. Birnbaum A. S. Kubik M. Y. Dishman R. K. (2004). Naturally occurring changes in physical activity are inversely related to depressive symptoms during early adolescence. Psychosom. Med. 66, 336–342. doi: 10.1097/01.psy.0000126205.35683.0a, 15184692

[ref64] Neece C. L. Green S. A. Baker B. L. (2012). Parenting stress and child behavior problems: a transactional relationship across time. Am. J. Intellect. Dev. Disabil. 117, 48–66. doi: 10.1352/1944-7558-117.1.48, 22264112 PMC4861150

[ref65] Öhman A. Nilsson S. Beckung E. (2010). Stretching treatment for infants with congenital muscular torticollis: physiotherapist or parents? A randomized pilot study. PM R 2, 1073–1079. doi: 10.1016/j.pmrj.2010.08.008, 21145518

[ref66] Orth H. Surowińska J. Żukrowski P. (2018). Terapia Metodą Vojty. Wrocław: Edra Urban & Partner.

[ref67] Pastor-Cerezuela G. Fernández-Andrés M. I. Tárraga-Mínguez R. Navarro-Peña J. M. (2016). Parental stress and ASD. Focus Autism Dev. Disabil. 31, 300–311. doi: 10.1177/1088357615583471

[ref68] Peplow U. C. Carpenter C. (2013). Perceptions of parents of children with cerebral palsy about the relevance of, and adherence to, exercise programs: a qualitative study. Phys. Occup. Ther. Pediatr. 33, 285–299. doi: 10.3109/01942638.2013.773954, 23477291

[ref69] Petry K. Maes B. (2007). Description of the support needs of people with profound multiple disabilities using the 2002 AAMR system: an overview of literature. Educ. Train. Dev. Disabil. 42, 130–143. doi: 10.1177/215416470704200203

[ref70] Prajzner A. (2022). Selected indicators of effect size in psychological research. Ann. Univ. Mariae Curie-Skłodowska J. Paedagog. Psychol. 35, 139–157. doi: 10.17951/j.2022.35.4.139-157

[ref71] Repka I. B. Betka P. Kuźmicz I. Puto G. Zurzycka P. (2019). Fatigue among parents caring for a child with cancer. Palliat. Med. 11, 88–96. doi: 10.5114/pm.2019.86626

[ref72] Roșca A. Sandu O. Rusu L. (2022). Vojta and Bobath combined treatment in the rehabilitation of balance in children with cerebral palsy. J. Sport Kinet. Mov. 1:38. doi: 10.52846/jskm/39.2022.1.5

[ref73] Rosnow R. L. Rosenthal R. (2003). Effect size for experimenting psychologists. Can. J. Exp. Psychol. 57, 221–237. doi: 10.1037/h0087427, 14596479

[ref74] Sadowska L. (2001). “Rozwój dziecka. Podstawy anatomiczne i patofizjologiczne,” in Neurokinezjologiczna Diagnostyka i Terapia Dzieci z Zaburzeniami Rozwoju Psychoruchowego, ed. Sadowska L. (Wrocław: Wydawnictwo AWF).

[ref75] Sadowska L. Krefft A. Wiraszka A. (2001). “Ocena diagnostyki i stymulacji metodą Vojty dzieci z zaburzeniami rozwoju psychomotorycznego,” in Neurokinezjologiczna Diagnostyka i Terapia Dzieci z Zaburzeniami Rozwoju Psychomotorycznego, ed. Sadowska L. (Wrocław: Wydawnictwo AWF).

[ref76] Sakkalou E. Sakki H. O’Reilly M. A. Salt A. T. Dale N. J. (2018). Parenting stress, anxiety, and depression in mothers with visually impaired infants: a cross-sectional and longitudinal cohort analysis. Dev. Med. Child Neurol. 60, 290–298. doi: 10.1111/dmcn.13633, 29219173

[ref77] Samadi S. A. Abdollahi-Boghrabadi G. McConkey R. (2020). Parental satisfaction with caregiving among parents of children with autism spectrum disorders, attention deficit and hyperactivity, intellectual disabilities and typically developing. Early Child Dev. Care 190, 1115–1122. doi: 10.1080/03004430.2018.1518903

[ref78] San-Martín-Gómez A. Cano-de-la-Cuerda R. Jiménez-Antona C. Viana-Meireles L. G. Salcedo-Perez-Juana M. Pérez-Corrales J. . (2026). “Demanding, but worth it”: the parental experience of home-based Vojta therapy for children presenting global developmental delay—a qualitative study using photo-elicitation. J. Clin. Med. 15:45. doi: 10.3390/jcm15010045, 41517295 PMC12786593

[ref79] Scherer N. Verhey I. Kuper H. (2019). Depression and anxiety in parents of children with intellectual and developmental disabilities: a systematic review and meta-analysis. PLoS One 14:e0219888. doi: 10.1371/journal.pone.0219888, 31361768 PMC6667144

[ref80] Schuch F. B. Vancampfort D. Firth J. Rosenbaum S. Ward P. B. Silva E. S. . (2018). Physical activity and incident depression: a meta-analysis of prospective cohort studies. Am. J. Psychiatry 175, 631–648. doi: 10.1176/appi.ajp.2018.17111194, 29690792

[ref81] Schuch F. B. Vancampfort D. Richards J. Rosenbaum S. Ward P. B. Stubbs B. (2016). Exercise as a treatment for depression: a meta-analysis adjusting for publication bias. J. Psychiatr. Res. 77, 42–51. doi: 10.1016/j.jpsychires.2016.02.023, 26978184

[ref82] Shilubane H. Mazibuko N. (2020). Understanding autism spectrum disorder and coping mechanisms by parents: an explorative study. Int. J. Nurs. Sci. 7, 413–418. doi: 10.1016/j.ijnss.2020.08.003, 33195753 PMC7644554

[ref83] Shokoohi-Yekta M. Ghobary-Bonab B. Malayeri S. A. Zamani N. Pourkarimi J. (2015). The relationship between anger and coping strategies of mothers of children with special needs. Procedia Soc. Behav. Sci. 205, 140–144. doi: 10.1016/j.sbspro.2015.09.041

[ref84] Sloan C. J. Mailick M. R. Hong J. Ha J. H. Greenberg J. S. Almeida D. M. (2020). Longitudinal changes in well-being of parents of individuals with developmental or mental health problems. Soc. Sci. Med. 264:113309. doi: 10.1016/j.socscimed.2020.113309, 32858491 PMC7441882

[ref85] Sowa J. (2004). “Biofizjologiczne podłoże procesu rehabilitacji,” in Człowiek z Niepełnosprawnością Intelektualną, ed. Janiszewska-Nieścioruk Z. (Kraków: Oficyna Wydawnictwa Impuls).

[ref86] Strojek K. Wójtowicz D. Kowalska J. (2022). Assessment of the emotional state of parents of children starting the Vojta therapy in the context of the physical activity: a pilot study. Int. J. Environ. Res. Public Health 19:10691. doi: 10.3390/ijerph191710691, 36078406 PMC9517770

[ref87] Takuri B. S. (2017). Stress and coping mechanism among the parents of intellectual disable children. J. Adv. Acad. Res. 1, 56–63. doi: 10.3126/jaar.v1i2.16589

[ref88] Theule J. Wiener J. Tannock R. Jenkins J. M. (2013). Parenting stress in families of children with ADHD: a meta-analysis. J. Emot. Behav. Disord. 21, 3–17.

[ref89] van de Mortel T. F. (2008). Faking it: social desirability response bias in self-report research. Aust. J. Adv. Nurs. 25, 40–48.

[ref90] van der Lubbe A. Swaab H. Vermeiren R. van Rossum E. van Balkom I. Ester W. A. (2025). Chronic parenting stress in parents of children with autism: associations with chronic stress in their child and parental mental and physical health. J. Autism Dev. Disord. doi: 10.1007/s10803-025-06736-9, 39982675 PMC13346221

[ref91] Verhaegh J. Nuijen M. (2022). Parents’ experiences with a home-based upper limb training program using a video coaching approach for infants and toddlers with unilateral cerebral palsy: a qualitative interview study. BMC Pediatr. 22:3432. doi: 10.1186/s12887-022-03432-w, 35768858 PMC9245237

[ref92] Vernhet C. Dellapiazza F. Blanc N. Cousson-Gélie F. Miot S. Roeyers H. . (2019). Coping strategies of parents of children with autism spectrum disorder: a systematic review. Eur. Child Adolesc. Psychiatry 28, 747–758. doi: 10.1007/s00787-018-1183-3, 29915911

[ref93] Vernhet C. Michelon C. Dellapiazza F. Rattaz C. Geoffray M. M. Roeyers H. . (2022). Perceptions of parents of the impact of autism spectrum disorder on their quality of life and correlates: comparison between mothers and fathers. Qual. Life Res. 31, 1499–1508. doi: 10.1007/s11136-021-03045-3, 34822048

[ref94] Vojta V. (1970). Reflex rotation as a pathway to human locomotion. Z. Orthop. Ihre Grenzgeb. 108, 446–452, 4250065

[ref95] Vojta V. Peters A. Gantner J. (2006). Metoda Vojty: Gry Mięśniowe w Odruchowej Lokomocji i w Ontogenezie Ruchu. Wrocław: Fundacja Promyk Słońca.

[ref96] Wójtowicz D. Skrzek A. Pyzio M. Żak M. (2007). Assessment of the Vojta therapy by parents as the therapists. Acta Bio-Opt. Inform. Med. 13, 300–304.

[ref97] Wong V. McGrew J. Ruble L. (2020). Predicting the outcomes of parents of transition-age youth or young adults with ASD. J. Autism Dev. Disord. 50, 2723–2739. doi: 10.1007/s10803-020-04362-1, 32030578

[ref98] Woodman A. C. Hauser-Cram P. (2013). The role of coping strategies in predicting change in parenting efficacy and depressive symptoms among mothers of adolescents with developmental disabilities. J. Intellect. Disabil. Res. 57, 513–530. doi: 10.1111/j.1365-2788.2012.01555.x, 22563652

[ref99] Xu T. Zhang Y. (2025). Different supports, different effects: social support moderates the impact of parenting stress on the mental health of parents of individuals with intellectual and developmental disabilities. Eur. J. Investig. Health Psychol. Educ. 15:248. doi: 10.3390/ejihpe15120248, 41440154 PMC12731844

